# Synthesis, Crystal Structure and Quantum Chemical Study on 3-Phenylamino-4-Phenyl-1,2,4-Triazole-5-Thione

**DOI:** 10.3390/molecules14020608

**Published:** 2009-02-04

**Authors:** Hong-Yan Wang, Pu-Su Zhao, Rong-Qing Li, Su-Min Zhou

**Affiliations:** 1 Jiangsu Key Laboratory for Chemistry of Low-Dimensional Materials Huaiyin Teachers College, Huaian, Jiangsu, 223300, P. R. China; E-mails: why6952@163.com (H-Y. W.), rongqingli333@yahoo.com (R-Q. L.); 2 Department of Chemical Engineering, Huaiyin Industry College, Huaian Jiangsu, 223001, P. R. China; E-mail: zhaopusu@163.com (S-M. Z.)

**Keywords:** Synthesis, Crystal structure, DFT, Second order optical nonlinearity.

## Abstract

3-Phenylamino-4-phenyl-1,2,4-triazole-5-thione was synthesized and characterized by elemental analysis, IR and X-ray single crystal diffraction. Density functional theory calculations of the structure, natural bond orbitals, atomic charge distributions and thermodynamic functions of the title compound were performed at B3LYP/ 6-311G** and PBE1PBE/6-311G**levels of theory, respectively. NPA atomic charge distributions indicate that the title compound can be used as a potential multi-dentate ligand to coordinate with various metallic ions. Calculation of the second order optical nonlinearity was also carried out. The thermodynamic properties of *C*^0^*_p,m_*, *S*^0^*_m_* and *H*^0^*_m_* were calculated and correlative equations between the thermodynamic properties and temperatures were also obtained.

## Introduction

Compounds containing a 1*H*-1,2,4-triazole group and its derivatives are of great interest because they exhibit fungicidal and plant-growth regulating activity [1,2]. They also show antibacterial activity against *Puccinia recondite* and root-growth regulation in cucumber [3]. In previous papers [4,5,6,7,8], we have reported several new compounds containing the 1,2,4-triazole moiety. 1,2,4-Triazole-5-thiones are important triazole derivatives and many mono-, di- and tri-substituted 1,2,4-triazole-5-thiones have been reported [9,10,11,12], but, to our knowledge, no structural data obtained either by experimental or theoretical methods have been reported so far for the title compound, 3-phenylamino-4-phenyl-1,2,4-triazole-5-thione. Especially, there have been no reports on the bioactivities of this compound till now, so we have synthesized the title compound and determinations of its bioactivities are currently in progress. On the other hand, in recent years, density functional theory (DFT) has become an increasingly useful tool for experimental studies. The success of DFT is mainly due to the fact that it describes small molecules more reliably than Hartree-Fock theory. It is also computationally less demanding than wave function based methods with inclusion of electron correlation [13,14]. Thus, in order to characterize the correlation between molecular structure and macroscopic properties in the studied compound, it seem to be essential to undertake a detailed comparative study of the isolated molecule and the solid state unit. In this paper, a concerted approach by X-ray crystallography and DFT calculation was used, which takes advantage of both the high interpretative power of the theoretical studies and the precision and reliability of the experimental method. At the same time, a comparison of some calculated results is also made with those of a similar compound, 3-benzyl-4-phenyl-1,2,4-triazole-5-thione, which was reported earlier by this group [8].

## Results and Discussion

### Description of the crystal structure

**Figure 1:**
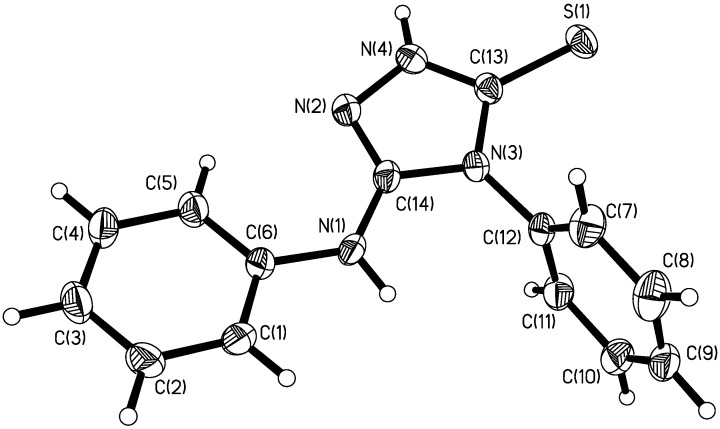
Molecular structure with the atomic numbering scheme for the title compound.

**Figure 2:**
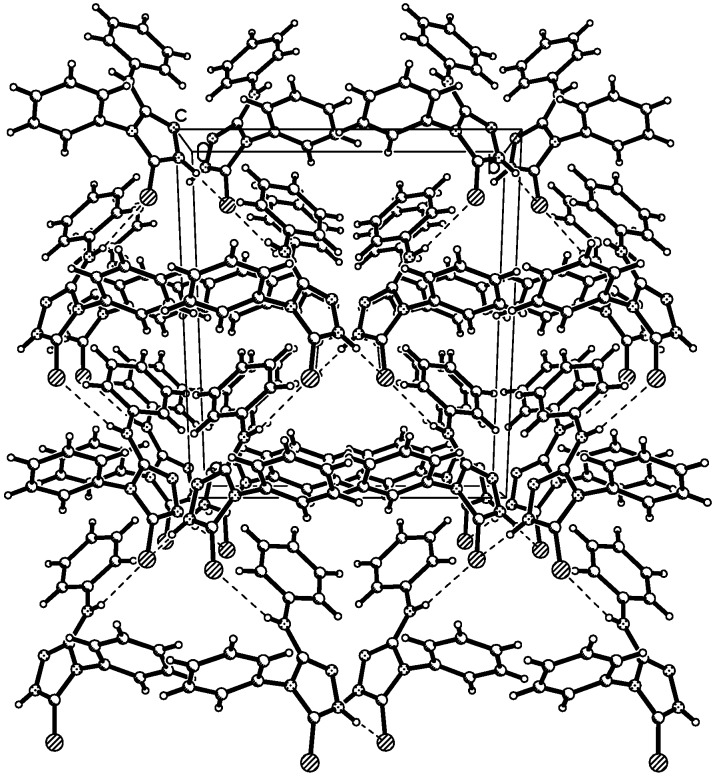
A view of the crystal packing down the *c* axis for the title compound.

The displacement ellipsoid plot with the numbering scheme for the title compound is shown in Figure 1. Figure 2 shows a perspective view of the crystal packing in the unit cell. Selected bond lengths and bond angles by X-ray diffractions are listed in Table 1, along with the calculated bond parameters.

**Table 1:** Selected structural parameters by X-ray and theoretical calculations.

Bond lengths (Å)	Exp.	B3LYP/6-311G**	PBE1PBE/6-311G**	Bond lengths (Å)	Exp.	B3LYP/6-311G**	PBE1PBE/6-311G**
S(1)-C(13)	1.692(3)	1.6684	1.659	N(1)-C(14)	1.339(4)	1.3644	1.3581
N(1)-C(6)	1.424(4)	1.4073	1.3985	N(2)-C(14)	1.301(4)	1.3034	1.2995
N(2)-N(4)	1.383(4)	1.3822	1.3683	N(3)-C(13)	1.365(4)	1.3985	1.391
N(3)-C(14)	1.388(4)	1.3924	1.3844	N(3)-C(12)	1.433(4)	1.431	1.4204
N(4)-C(13)	1.313(4)	1.3498	1.3439	C(1)-C(2)	1.374(5)	1.3882	1.3846
C(3)-C(4)	1.362(5)	1.3916	1.388	C(5)-C(6)	1.374(5)	1.3998	1.3959
C(7)-C(12)	1.361(5)	1.394	1.3904	C(8)-C(9)	1.378(6)	1.3941	1.3907
C(9)-C(10)	1.350(6)	1.3931	1.3894	C(11)-C(12)	1.370(4)	1.3947	1.3918
Bond angle (º)				Bond angle (º)			
C(13)-N(3)-C(14)	107.6(3)	107.7899	107.7477	C(14)-N(2)-N(4)	103.0(3)	103.2568	103.2951
C(13)-N(4)-N(2)	114.6(3)	115.1305	115.2874	C(13)-N(3)-C(12)	127.4(3)	125.9714	125.993
N(4)-C(13)-N(3)	103.9(3)	102.0667	102.0046	C(7)-C(12)-N(3)	119.2(3)	119.6807	119.7079
C(5)-C(6)-N(1)	123.6(3)	123.3155	123.3114	C(14)-N(1)-C(6)	127.0(3)	129.0576	128.8431
C(4)-C(3)-C(2)	119.0(3)	119.0961	119.0674	C(2)-C(1)-C(6)	120.6(3)	120.3618	120.3537
C(10)-C(9)-C(8)	120.5(4)	120.146	120.1041	C(6)-C(5)-C(4)	119.0(3)	119.3328	119.2883
C(12)-C(11)-C(10)	119.1(4)	119.4385	119.4273	C(12)-C(7)-C(8)	119.3(4)	119.3845	119.2746
N(4)-C(13)-S(1)	128.4(3)	129.069	129.0636	N(2)-C(14)-N(1)	127.8(3)	128.1275	128.2003

The molecular structure of the title compound consists of discrete [PhNHC_2_N_3_HSPh] entities. All of the bond lengths and bond angles in the phenyl rings are in the normal range. The C=S bond length of 1.692(3) Å is comparable to that found in 3-benzyl-4-phenyl-1,2,4-triazole-5-thione [C=N 1.687(3) Å] [8]. In the triazolyl ring, the N-N, C=N and C-N bond lengths are also in good agreement with those found in the above-cited structure [8]. The exocyclic sulfur atom and triazolyl ring define a plane *P*1. The dihedral angles between *P*1 with phenylamino planar and another phenyl ring are 16.98(2) and 83.36(1)°, respectively. The dihedral angle between the phenyl ring and phenylamino planar is 80.56(3)°.

In the crystal lattice, there are two intermolecular interactions, one potentially weak intramolecular interaction (C-H···Y, Y= N)[15,16] and some C-H···*π* supramolecular interactions (see Table 2) [17]. In the solid state, all above supramolecular interactions stabilize the crystal structures.

**Table 2:** Hydrogen bonds and C-H••• *π* supramolecular interactions.

D-H•••A	Symmetry	D•••A (Å)	∠D-H•••A (°)
N(1)-H(1A)•••S(1)	-1/2+*x*,1/2-*y*,-1/2+*z*	3.6963	159.75
N(4)-H(4A)•••S(1)	*x*, 1-*y*, -1/2+*z*	3.2696	139.09
C(5)-H(5A) ••• N(2)		2.9298	122.23
C(2)-H(2B)•••Cg(1) [triazolyl ring]	-1/2*+x,* 1/2*-y,* -1/2+*z*	3.747	108.59
C(3)-H(3A)•••Cg(3)[C(7)-C(12)]	-1/2+*x* ,1/2-*y,* -3/2+ z	3.685	164.73
C(5)-H(5A)•••Cg(1) [triazolyl ring]	*x,* 1-*y,* -1/2+ *z*	3.944	134.62
C(8)-H(8A) •••Cg(2)[C(1)-C(6)]	1/2+*x,* -1/2+*y,* 1+ *z*	4.035	142.60
C(10)-H(10A)•••Cg(2)[C(1)-C(6)]	*x , y, 1+ z*	3.562	148.46

### Optimized geometry

DFT calculations were performed on the title compound at the B3LYP/6-311G** and PBE1PBE/6-311G** levels of theory. Some optimized geometric parameters are also listed in Table 1. In view of the bond lengths in Table 1, most predicted values are longer than experimental ones and the biggest difference between the theoretical and experimental values occurs at C(9)-C(10) bond, with the different values being 0.0431Å for B3LYP method and 0.0394 Å for PBE1PBE method. As for the bond angles, most predicted values are corresponding with the experimental values and the biggest difference takes place at bond angle of C(14)-N(1)-C(6), with the different values being 2.0576º for B3LYP method and 1.8431º for PBE1PBE method. The reasons for above discrepances maybe as follows: (1) the theoretical values belong to the isolated molecule in gas-phase and the experimental values are attributed to the molecule in solid state. The geometry of the solid-state structures is subject to intermolecular forces, such as van der Waals interactions and crystal packing forces, which make most of the experimental bond lengths be shorter than the theoretical ones; (2) in the solid states, there exists an intermolecular hydrogen-bond interaction corresponding with N(1)-H(1) bond, while in theoretical calculations, intermolecular interactions are neglected, which, to some extent, may lead to the bond angle of C(14)-N(1)-C(6) smaller in solid than that in calculations. Despite of some differences, both DFT methods used here can reproduce the molecular geometry on the whole and they are the bases for our following discussion.

### Natural bond orbital (NBO) analysis

The NBO occupancies based on the B3LYP/6-311G** and PBE1PBE/6-311G** optimized structures are calculated and listed in Table 3. As seen from this data, the following four conclusions can be drawn: (1) the N(2)-C(14) bond is a normal double–bond and, in two phenyl rings, the C–C bonds are in typical single-double arrangements, which form the conjugate structure and support the information in crystal structure. The similar situation can be found in the compound of 3-benzyl-4-phenyl-1,2,4-triazole-5-thione [8]; (2) the N(3)-C(12) bond is only a single bond, which is different from that seen in 3-benzyl-4-phenyl-1,2,4-triazole-5-thione [8], where N(3)-C(12) has some double-bond character; (3) in B3LYP calculations, the S(1)-C(13) bond represents a double bond, which is in good agreement with the experimental fact, while in PBE1PBE calculations, the S(1)-C(13) bond only has single bond character; (4) except for the N(2)-N(4) bond, all of the single bonds, including the C-C single bonds in the two phenyl rings, along with the double bond of N(2)-C(14) in PBE1PBE calculations have smaller occupancies than those in B3LYP calculations. In a word, to some extent, for the system studied here, apart from the bond of S(1)-C(13), NBO results obtained by B3LYP/6-311G**and PBE1PBE/6-311G** both correspond to the experiments.

**Table 3:** Natural bond orbital occupancies^*^.

Bond	Occupancies(a.u)		Bond	Occupancies(a.u)	
	B3LYP/6-311G**	PBE1PBE/6-311G**		B3LYP/6-311G**	PBE1PBE/6-311G**
S(1)-C(13)	1.99384	1.98157	C(3)-C(4)π	1.36614	1.67131
S(1)-C(13)π	1.98170		C(3)-C(4)π*	0.33789	0.33783
S(1)-C(13)π*	0.56667		C(4)-C(5)	1.97530	1.97504
N(3)-C(13)	1.97620	1.97596	C(5)-C(6)	1.97189	1.97129
N(3)-C(14)	1.97569	1.97544	C(5)-C(6)π	1.64608	1.64588
N(4)-C(13)	1.98882	1.98854	C(5)-C(6)π*	0.39220	0.39257
N(2)-N(4)	1.97738	1.97787	C(7)-C(12)	1.97212	1.97113
N(2)-C(14)	1.98267	1.98223	C(7)-C(8)	1.97552	1.97532
N(2)-C(14)π	1.92656	1.92444	C(7)-C(8)π	1.64506	1.64350
N(2)-C(14)π*	0.39694	0.39702	C(7)-C(8)π*	0.30839	0.30585
N(1)-C(14)	1.98381	1.98355	C(8)-C(9)	1.97949	1.97941
N(1)-C(6)	1.98431	1.98387	C(9)-C(10)	1.97945	1.97930
N(3)-C(12)	1.98196	1.98143	C(9)-C(10)π	1.65212	1.65422
C(1)-C(6)	1.97048	1.96976	C(9)-C(10)π*	0.31849	0.31979
C(1)-C(2)	1.97622	1.97617	C(10)-C(11)	1.97542	1.97514
C(1)-C(2)π	1.70572	1.70603	C(11)-C(12)	1.97228	1.97152
C(1)-C(2)π*	0.34018	0.33989	C(11)-C(12)π	1.68578	1.68803
C(2)-C(3)	1.97847	1.97838	C(11)-C(12)π*	0.37620	0.38247
C(3)-C(4)	1.97940	1.97909			

^*^ anti-bond

### Atomic charge distributions

Based on B3LYP/6-311G** and PBE1PBE/6-311G** optimized geometries, Natural Population Analysis (NPA) atomic charge distributions were calculated. All of the atomic charges of non-hydrogen atoms are listed in Table 4.

**Table 4:** NPA atomic charge distributions obtained at B3LYP/6-311G** and PBE1PBE/6-311G** levels.

Atom	Charges (*e*)		Atom	Charges (*e*)		Atom	Charges (*e*)	
	B3LYP	PBE1PBE		B3LYP	PBE1PBE		B3LYP	PBE1PBE
S(1)	-0.25706	-0.25839	C(1)	-0.23800	-0.24608	C(7)	-0.17805	-0.18447
N(2)	-0.37039	-0.36728	C(2)	-0.18199	-0.18736	C(8)	-0.18504	-0.19000
N(3)	-0.46577	-0.45806	C(3)	-0.22327	-0.23177	C(9)	-0.18313	-0.19100
N(4)	-0.37746	-0.37514	C(4)	-0.17350	-0.17946	C(10)	-0.18927	-0.19689
C(13)	0.23522	0.23106	C(5)	-0.24558	-0.25464	C(11)	-0.19869	-0.22115
C(14)	0.59570	0.58960	C(6)	0.17109	0.16826	C(12)	0.12351	0.12458
N(1)	-0.59232	-0.59554						

As seen from Table 4, since the nitrogen and sulfur atoms have greater electronegativity than the carbon and hydrogen atoms, all of the N atoms and the S atom carry negative charges. Although N(1), N(3) and N(4) have bigger negative charges, steric effects around them hinder these atoms from coordinating further with metallic ions. On the other hand, the negative charges on N(2) and S(1) are lesser than those on N(1), N(3) and N(4), however, the expansive environment around them make these two atoms potential reaction sites for metallic ions, which suggest that the title compound can be used as a terminal or multidentate ligand to coordinate with various metallic ions and thus assemble diverse metal complexes. This is not only consistent with many experimental facts reported before [18,19], but also supports one of our original aims for the synthesis.

### Calculation of nonlinear optical properties

On the basis of the MNDO Hamiltonian and PM3 parametrization with the MOPAC program package, the molecular hyperpolarizability value, βμ, the vector components along the dipole moment direction, of the title compound were calculated to be 7.166×10-30 esu, which is greater than those for urea (0.14×10-30 esu calculated using the same method) [20] and para-nitroaniline (PNA) (6.801×10-30 esu calculated using the same method). The βμ, value of the title compound is also larger than those of similar compounds 4-phenyl-3-[(1,2,4-triazol-1-yl)methyl]-triazole-5-thione (4.397×10-30esu) [21] and 3-benzyl-4-phenyl-1,2,4-triazole-5-thione (4.025×10-30 esu) [8]. Based on the optimized geometry at the B3LYP/6-311G** level of theory, the dipole moment of the title compound was calculated, which was 5.2638 Debye. Using the TD-DFT method, the electronic spectra of the title compound were predicted and two transition bands were obtained, one being at 246.92 nm, with an oscillator strength *f* = 0.3151 and another at 229.23 nm, with oscillator strength *f* = 0.2900. Both electronic transition bands are in the ultraviolet region, showing the good transparency of the title compound. These calculations indicate that the title compound might be a good candidate as a second-order nonlinear optical material.

### Thermodynamic propertes

On the basis of B3LYP/6-311G** and PBE1PBE/6-311G** vibrational analyses and statistical thermodynamics, the standard thermodynamic functions: heat capacity (*C*^0^*_p,m_*), entropy (*S*^0^*_m_*) and enthalpy (*H*^0^*_m_*) of the title compound were obtained and are listed in Table 5. The scale factor for frequencies was 0.96. For 3-benzyl-4-phenyl-1,2,4-triazole-5-thione, the corresponding value of *C*^0^*_p,m_* , *S*^0^*_m_* and *H*^0^*_m_* obtained at B3LYP/6-311G** level of theory were also listed in Table 5.

**Table 5:** Thermodynamic properties for the title compound and 3-benzyl-4-phenyl-1,2,4-triazole-5-thione [8].

	The title compound						3-Benzyl-4-phenyl-1,2,4-triazole-5-thione		
	B3LYP/6-311G**			PBE1PBE/6-311G**			B3LYP/6-311G**		
*T/*K	*C*^0^*_p,m_*^†^	*S*^0^*_m_*^†^	*H*^0^*_m_*^‡^	*C*^0^*_p,m_*^†^	*S*^0^*_m_*^†^	*H*^0^*_m_*^‡^	*C*^0^*_p,m_*^†^	*S*^0^*_m_*^†^	*H*^0^*_m_*^‡^
100.0	110.75	368.74	7.61	109.37	361.77	7.43	110.23	369.32	7.68
200.0	187.75	468.16	22.39	185.89	460.10	22.06	184.54	467.31	22.24
298.1	272.63	558.90	44.98	270.04	549.96	44.42	270.94	556.94	44.56
300.0	274.21	560.59	45.48	271.60	551.64	44.92	272.58	558.62	45.06
400.0	354.11	650.71	77.00	351.00	640.93	76.15	355.90	648.73	76.59
500.0	419.70	737.05	115.82	416.47	726.56	114.65	424.90	735.85	115.76
600.0	471.45	818.34	160.48	468.34	807.26	158.99	479.58	818.34	161.09
700.0	512.41	894.20	209.75	509.50	882.66	207.96	522.99	895.65	211.30

^†^ J·mol^-1^·K^-1^; ^‡^ kJ·mol^-1^

As observed from Table 5, all the values of *C*^0^*_p,m_*, *S*^0^*_m_* and *H*^0^*_m_* increase with the increase of temperature from 100.0 to 700.0 K, which is attributed to the enhancement of the molecular vibration as the temperature increases. On the other hand, for the title compound, all of the values of *C*^0^*_p,m_* , *S*^0^*_m_* and *H*^0^*_m_* obtained by the PBE1PBE/6-311G** method are smaller than the corresponding values obtained by the B3LYP/6-311G** method. Thirdly, compared with 3-benzyl-4-phenyl-1,2,4-triazole-5-thione, the phenylamino group at the 3-position of the triazolyl ring of the title compound has a higher molecular weight than that of the benzyl group of 3-benzyl-4-phenyl-1,2,4-triazole-5-thione, however, the *C*^0^*_p,m_* , *S*^0^*_m_* and *H*^0^*_m_* values at each temperature obtained at the B3LYP/6-311G** level for the title compound are not always lager than those of 3-benzyl-4-phenyl-1,2,4-triazole-5-thione [8]. The correlation equations between these thermodynamic properties and temperatures for the title compound, are listed in Table 6.

**Table 6:** The correlation equations between thermodynamic properties and temperature.

B3LYP/6-311G**	*C*^0^*_p,m_* = 4.722 + 1.042 *T* – 4.443*10 ^-4^*T ^2^* ( *R*^2^ = 0.9987 )
	*S*^0^*_m_* = 266.297 + 1.047 *T* – 2.135*10 ^-4^*T ^2^* ( *R*^2 ^= 0.9999)
	*H*^0^_m_ = -2.665 + 5.726*10^-2 ^ *T* + 3.542*10^-4^*T ^2^*( *R*^2 ^= 0.9998 )
PBE1PBE/6-311G**	*C*^0^*_p,m_* = 4.434 + 1.031 *T* – 4.342*10 ^-4^*T ^2^* ( *R*^2^ = 0.9988 )
	*S*^0^*_m_* = 260.483 + 1.035 *T* –2.077*10 ^-4^*T ^2^* ( *R*^2 ^= 0.9999)
	*H*^0^_m_ =-2.649 + 5.573*10^-2 ^*T* + 3.527*10^-4^*T ^2^*( *R*^2 ^= 0.9998 )

These equations could be used for the further studies on the title compound. For instance, when we investigate the interaction between the title compound and another compound (for example DNA molecules ), thermodynamic properties *C*^0^*_p,m_* , *H*^0^*_m_* and *S*^0^*_m_* could be obtained from these equations and then used to calculate the change of Gibbs free energy of the reaction, which will assist us to judge the spontaneity of the reaction.

### Biological activity

The primary biological test has shown that the title compound exhibits inhibiting activity towards some fungi, such as *P. zeae, A. solani* and *P. piricola*, with inhibition rates by the agar plate diffusion method [22] of 24.1%，24.1% and 45.0% at 50 μg/mL, respectively.

## Experimental and Theoretical Methods

### General

Elemental analyses for carbon, hydrogen and nitrogen were performed on a Perkin-Elmer 240C instrument. IR spectra (4000–400 cm^−1^), as KBr pellets, were recorded on a Nicolet FT-IR spectrophotometer. All chemicals were obtained from a commercial source (J&K Chemical Ltd., Beijing, P.R. China) and used without further purification. ^1^H-NMR spectra were recorded on a Bruker model DRX 500 spectrometer in CDCl_3_.

### Synthesis

**Scheme 1:**
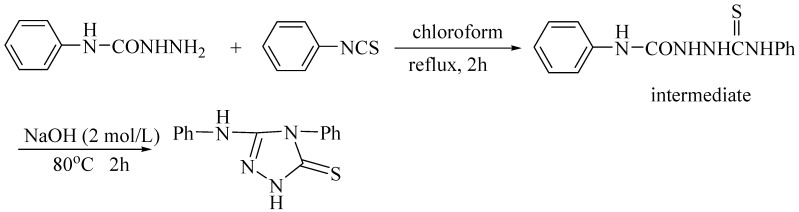
Synthesis of the title compound

The title compound was synthesized in two steps as shown in Scheme 1. 

First, equimolar amounts of phenylaminoformyl hydrazide and phenyl isothiocyanate were mixed in chloroform with refluxed to give, after 2 h, white solids which were recrystallized from a mixture of DMF-C_2_H_5_OH-water to afford afforded the intermediate, of which 5 mmol was added to 2 mol/L NaOH solution (15 mL) and stirred until the intermediate dissolved. Then, the mixture was heated at 80^o^C for 2 h and then cooled to room temperature. After the mixture was adjusted to pH = 7 using 10% hydrochloric acid, a great deal of white solid was observed. The title compound was obtained by recrystallization the white solid from the mixture of DMF and C_2_H_5_OH. Yield 95%. Mp.178-179 ^o^C. IR: *ν* 3487 (m), 3067 (m), 1604 (vs), 1554 (s), 1525 (s), 1469 (m), 1446 (s), 1340 (m), 1325 (m), 1237 (m), 1207 (m), 1162 (vs), 1083 (m), 1048 (m), 1029 (m), 1011 (w), 996 (m), 872 (s), 842 (vs), 745 (s), 699 (s), 637 (m), 560 (m) cm^−1^. ^1^H-NMR: 4.0 (s, 1H, -**H**N-Ph), 6.43-6.67 and 7.0-7.04 (m, 11H, two C_6_**H**_5_- and C_2_N_3_S**H**-); Found: C, 62.41; H, 4.38; N, 20.72%.Calc. for C_14_H_12_N_4_S: C, 62.66, H, 4.51, N, 20.88%.

### Crystallographic study

The selected crystal of the title compound was mounted on an Enraf-Nonius CAD4 diffractometer. Reflection data were measured at 20°C using graphite monochromated Mo-K*α* (*λ* = 0.71073 Å ) radiation and a *ω*-2*θ* scan mode. The correction for *Lp* factors and empirical absorption were applied to the data. The structures were solved by direct methods and refined by full-matrix least-squares method on *F*_obs_^2^ using the SHELXTL software package [23]. All non-H atoms were anisotropically refined. The hydrogen atom positions were fixed geometrically at calculated distances and allowed to ride on the parent C atoms. The final least-square cycle gave *R* = 0.0463, *wR*_2_ = 0.0980. Atomic scattering factors and anomalous dispersion corrections were taken from International Table for X-ray Crystallography [24]. A summary of the key crystallographic information is given in Table 7.

CCDC-649123 contains the supplementary crystallographic data for this paper. These data can be obtained free of charge at www.ccdc.cam.ac.uk/conts/ retrieving.html [or from the Cambridge CrystallographicData Centre (CCDC), 12 Union Road, Cambridge CB2 1EZ, UK; fax: +44(0)1222-336033; email: deposit@ccdc.cam.ac.uk].

**Table 7:** Summary of crystallographic results for the title compound.

Empirical formula	C_14_H_12_N_4_S
Formula weight	268.34
Temperature	293(2) K
Wavelength	0.71073 Å
Crystal system, space group	Monoclinic, *Cc*
Unit cell dimensions	*a* = 15.232(3) Å, *b* = 11.758(2) Å, *c* = 8.6025(17) Å, *β* = 121.66(3)°
Volume	1311.5(5) Å^3^
*Z*, Calculated density	4, 1.359 Mg/m^3^
Absorption coefficient	0.238 mm^-1^
*F*(000)	560
*θ* range for data collection	2.34 to 25.99°
Limiting indices	-18≤ *h* ≤ 18, -9 ≤ *k* ≤ 14, -10≤ *l* ≤10
Reflections collected / unique	2794 / 2221 [*R*_int _= 0.0223 ]
Refinement method	Full-matrix least-squares on *F*^2^
Data / restraints / parameters	2221 / 2 / 172
Goodness-of-fit on *F*^2^	1.075
Final *R* indices [*I*>2*σ* (*I*)]	*R*_1_ = 0.0463, *wR*_2_ = 0.0980
*R* indices (all data)	*R*_1_ = 0.0492, *wR*_2_ = 0.1006
Largest diff. peak and hole	0.203and -0.232e. Å^-3^

### Computational methods

In order to find out which method is more suitable to the system studied here, two density functional theory (DFT ) calculations at the B3LYP/6-311G** and PBE1PBE/6-311G** [25,26] levels of theory by the Berny method [26] were performed with the Gaussian 03 software package [28]. Vibrational frequencies calculated ascertain the structure was stable (no imaginary frequencies). Based on the statistical thermodynamics and vibrational analyses, the thermodynamic properties of the title compound at different temperatures were calculated. Natural Bond Orbital (NBO) analyses and the time–dependent density functional theory (TD-DFT) [29,30,31,32] calculations of electronic absorption spectra were also performed on the optimized structure. On the basis of the MNDO Hamiltonian [33] and PM3 parametrization [34] with the MOPAC [35] program package, the molecular hyperpolarizability value was also calculated. All calculations were performed on a DELL PE 2850 server and a Pentium IV computer using the default convergence criteria.
